# Peer Review of “Selection of the Optimal L-asparaginase II Against Acute Lymphoblastic Leukemia: An In Silico Approach“

**DOI:** 10.2196/33216

**Published:** 2021-09-08

**Authors:** Ariz Mohammad

**Affiliations:** 1 Department of Genetics Washington University in Saint Louis St. Louis, MO United States

*This is a peer-review report submitted for the paper “Selection of the Optimal L-asparaginase II Against Acute Lymphoblastic Leukemia: An In Silico Approach”.*

## Round 1 Review

### General Comments

L-Asparaginase II (asnB) derived from *E coli* and *E chrysanthemi* is often used in the treatment of acute lymphoblastic leukemia (ALL). The manuscript submitted by Baral et al [1] outlines an in silico method to identify potential asnB from different species with potentially higher potency against suppressing the tumor and lesser side effects. Using over 100 asnB from a wide range of species, the authors identified a group of potential candidates and have taken them up for further analysis. Using homology modeling, the structures of these candidate proteins were built and were then used to calculate binding energies with asparagine. The authors also showed that the predicted binding energies have an inverse relationship with the reported experimental Km values of asnBs. This led authors to predict 3 asnBs from 3 different Streptomyces species.

The manuscript has systematically presented the findings and is nicely assembled. I have a few concerns.

### Specific Comments

#### Major Comments

The figures need to be made compact and some should be combined into one (see below).

#### Minor Comments

1. Page 3, Introduction, line 2: Cite peer-reviewed article/review.
2. Page 3: Keep a space between text and citation parentheses.
3. Page 3, paragraph 2: First sentence is abrupt. Rewrite the paragraph, probably starting with the discovery of the guinea pig serum cure of ALL!
4. Page 5, line 1: Change “analyses” to analyze.
5. Figure 2: Should be rearranged and each plot should be labeled for species, and the unnecessary text should be removed like postscript file indicator, plot number, etc.
6. Figures 3-5b: Use an arrow to show the point.
7. Figures 3-5: Combine the three figures into one.
8. Figure 7: Figure needs to be combined and made compact. The insets are too big. Species name should be given on individual panels.
9. Figure 8: Figure needs to be combined and made compact.

## Abbreviations

ALL: acute lymphoblastic leukemia
